# Alzheimer’s Disease: From Pathogenesis to Emerging Therapeutic Targets

**DOI:** 10.3390/jcm15062357

**Published:** 2026-03-19

**Authors:** Tetsuya Takahashi, Kazuki Muguruma

**Affiliations:** 1 MNES Inc., 1-2-27 Shinonome Honmachi, Minami-ku, Hiroshima-shi 734-0023, Hiroshima, Japan; 2 Department of Rehabilitation, Faculty of Rehabilitation, Hiroshima International University, Kurose-Gakuendai 555-36, Higashihiroshima-shi 739-2695, Hiroshima, Japan; 3 Department of Neurology, Hiroshima City Rehabilitation Hospital, 1-39-1, Tomominami, Asaminami-ku, Hiroshima-shi 731-3168, Hiroshima, Japan; sixcars01@yahoo.co.jp

**Keywords:** APP/Aβ, tau, lysosome, APOE, gangliosides, v-ATPase, asparaginyl endopeptidase, microglia, LIPUS

## Abstract

Alzheimer’s disease (AD) is the most prevalent cause of dementia and can be conceptualized as a tauopathy initiated by the accumulation of amyloid-β (Aβ) in the brain. The clinical introduction of anti-Aβ antibody therapies has marked the beginning of a new era in disease-modifying treatment for dementia. While the deleterious effects of Aβ on postsynaptic spines and axonal microtubules have been increasingly clarified, recent studies have shifted attention beyond extracellular Aβ deposition as senile plaques to the pathogenic significance of intracellular Aβ. In particular, accumulating evidence highlights lysosomes as critical sites of intracellular Aβ toxicity. Interactions between Aβ and gangliosides, v-ATPase-dependent lysosomal acidification, and lysosomal membrane integrity are the key determinants of disease progression. In parallel, additional molecular players, including components of the complement cascade and asparaginyl endopeptidase, have been implicated in linking Aβ pathology to tau dysregulation and neurodegeneration. As therapeutic strategies targeting Aβ enter clinical practice, these emerging pathways represent promising targets for the next generation of AD treatment. Here, we summarize current insights and ongoing therapeutic developments centered on these mechanisms.

## 1. Introduction

The clinical implementation of antibody-based therapies has ushered in a new era of disease-modifying treatment for Alzheimer’s disease (AD). Accordingly, this mini-review focuses on AD, the most prevalent form of dementia. The advent of antibody therapies has not only established the amyloid hypothesis as the conceptual foundation for therapeutic development, but their clinical outcomes have also served to test and substantiate this hypothesis. Among these antibodies, aducanumab was withdrawn after launch owing to insufficient efficacy data, and gantenerumab was withdrawn after phase 3 trials due to deficient clinical efficacy [1]. Currently, lecanemab and donanemab are post-launch drugs; however, currently available anti-amyloid therapies often require repeated hospital visits, imposing a considerable burden on both patients and health-care systems. Given the increased risk of amyloid-related imaging abnormalities (ARIA) in APOE ε4 carriers [1], especially in homozygotes, careful patient selection and close MRI-based safety monitoring are warranted during treatment. Because these therapies act on upstream pathogenic processes, they are primarily indicated in the early stages of the disease. Nevertheless, even within this limited indication, the growing number of eligible patients raises concerns regarding the capacity of medical institutions to provide treatment. Recent advances in drug delivery, including the development of subcutaneous formulations, may reduce the burden associated with long-term treatment. However, as several anti-amyloid antibodies have already entered clinical practice, there is an increasing need to explore therapeutic strategies based on alternative mechanisms of action.

Accumulating evidence suggests that lysosomes are critical sites of amyloid-β (Aβ) processing and that lysosomal dysfunction plays an important role in the pathogenesis of AD. In this review, we deliberately refrained from discussing antibody therapy in detail. Instead, we highlight several emerging lines of evidence that may serve as the basis for next-generation therapeutic targets involving molecules that converge on endo-lysosomal function. By doing so, we aim to outline the broad contours of AD, a disease of extraordinary complexity that is often examined through isolated and partial perspectives.

## 2. Alzheimer’s Disease: A Brief Retrospective

The pathological hallmark of AD, now known as senile plaque, was referred to until the early twentieth century as drusige Nekrose or drusige Degeneration [2,3]. This terminology reflected a morphological interpretation of senile plaques as small nodular degenerative products and can be found not only in Alois Alzheimer’s original 1906 report but also in the 1910 work of his disciple, Gaetano Perusini [2,4]. Subsequently, the term senile plaque became established and has since been adopted as a standard neuropathological concept.

The term Druse itself originates from bacteriology. In the late nineteenth century, Israel described radiating aggregates of filamentous bacteria in infections caused by Actinomyces species and referred to these structures as Drusen [5]. This designation later became widely accepted as a defining morphological feature of Actinomyces.

In ophthalmology, the term druse was introduced even earlier. In 1856, nodular deposits located beneath the retinal pigment epithelium, characteristic of age-related macular degeneration (AMD), were named drusen because their appearance was reminiscent of mineral crystal aggregates [6]. To date, the presence, size, and distribution of drusen remain essential indicators for the diagnosis and prognostic assessment of AMD.

Notably, it has become clear that both senile plaques—formerly described as drusige Degeneration—and retinal drusen in AMD share amyloid-β (Aβ) as a major constituent [7]. This observation suggests that common molecular mechanisms may underlie age-related degenerative pathology in distinct tissues, such as the brain and retina, representing a compelling intersection between the historical evolution of terminology and modern molecular neuropathology.

More recently, non-invasive methods for detecting retinal Aβ deposition have been developed. Studies employing hyperspectral retinal imaging [8], as well as novel biomarkers based on spatial correlation analysis of optical coherence tomography signal intensity [9] have demonstrated the feasibility of directly visualizing retinal Aβ accumulation. These approaches raise the possibility that retinal imaging could be applied to the diagnosis and monitoring of AD.

## 3. Amyloid-β Oligomerization, Intracellular Accumulation, and Lysosomal Dysfunction in Alzheimer’s Disease

In the progression of AD, amyloid-β (Aβ) oligomerization and its subsequent accumulation within neurons play central pathogenic roles. The origins of AD research can be traced back to Alois Alzheimer’s original pathological descriptions of senile plaques and neurofibrillary changes in 1906 [4]. In the 1980s, the cloning of the amyloid precursor protein (APP) gene [10] and the identification of APP mutations as causes of familial AD (FAD) [11], together with the discovery that mutations in presenilin-1 and presenilin-2 (PSEN1/2) [12,13], which encode the catalytic components of the γ-secretase complex, can also cause AD, led to the formulation of the amyloid cascade hypothesis [14]. This hypothesis places Aβ at the top of the AD pathogenesis hierarchy.

Initially, extracellular amyloid plaques were considered the primary neurotoxic species in AD. However, subsequent studies have demonstrated that plaque burden correlates poorly with cognitive impairment, whereas soluble Aβ oligomers disrupt synaptic plasticity. A seminal study showed that naturally secreted Aβ oligomers inhibit hippocampal long-term potentiation in vivo [15], and accumulating evidence has established soluble oligomeric Aβ as the principal synaptotoxic entity [16].

Consistent with this shift, lecanemab, the first anti-Aβ antibody to demonstrate clinical efficacy, preferentially binds to Aβ oligomers and protofibrils rather than mature fibrils. The differences in the binding profiles of lecanemab, aducanumab, and gantenerumab may underlie the therapeutic efficacy and adverse effects observed in clinical trials [17]. In parallel, the small-molecule agent tramiprosate and its prodrug valiltramiprosate (ALZ-801), currently in Phase 3 clinical trials (APOLLOE4; NCT04770220), bind to Aβ42 monomers and prevent their conversion into toxic oligomeric and fibrillar species. Preclinical and clinical data suggest that ALZ-801 has disease-modifying potential by stabilizing non-toxic Aβ conformations [18,19].

Beyond extracellular deposition, it is now well established that Aβ accumulates intracellularly, particularly within endosomal and lysosomal compartments. Intraneuronal Aβ42 accumulation within multivesicular bodies was first demonstrated by Takahashi et al. [20], shifting the focus toward intracellular Aβ as a pathogenic driver of AD.

APP functions physiologically as an adhesion molecule linking pre- and postsynaptic membranes [21]; however, a substantial fraction undergoes endocytosis independent of its adhesive role. Following internalization, APP is sequentially cleaved by β- and γ-secretases along the endosomal–lysosomal pathway, generating Aβ peptides intracellularly. When γ-secretase activity is relatively high, trimming proceeds toward shorter peptides (Aβ37–40), whereas reduced γ-secretase activity favors the production of longer aggregation-prone Aβ42 and Aβ43 species. Using the ratio (Aβ37 + 38 + 40)/(Aβ42 + 43) of PSEN1 mutant cell lines as an index of γ-secretase activity, a strong positive correlation (R^2^ = 0.78) has been demonstrated between residual γ-secretase function of PSEN1 variants and age at disease onset [22].

Importantly, Yu et al. showed that Aβ can be generated directly from APP within lysosomal compartments, implicating lysosomes in AD pathogenesis from the earliest stages [23]. Nixon and colleagues reported that, in five different mouse models of AD, autolysosomes with relatively elevated luminal pH accumulated in the perinuclear region of neurons. These autolysosomes were found to contain APP-derived fragments, including βCTF and Aβ. Over time, autolysosomes progressively enlarged and contributed to the formation of plasma membrane blebs. Ultimately, the affected neurons develop a distinctive morphology resembling flower petals, a phenotype that the authors termed PANTHOS. Similar pathological features have been observed in the brains of patients with AD. Based on these observations, the authors proposed that intracellularly generated Aβ may be released into the extracellular environment following neuronal death, where it subsequently contributes to the formation of senile plaques [24].

Extracellular Aβ oligomers can also enter neurons via heparan sulfate proteoglycan-dependent macropinocytosis and lipid raft-mediated endocytosis, ultimately reaching the lysosomes of neighboring cells [25]. Thus, lysosomal Aβ accumulation arises from both intracellular production and extracellular uptake, creating a self-amplifying pathogenic cycle [26].

The lysosomal microenvironment profoundly influences Aβ metabolism. Lysosomal membranes contain lipid raft-like microdomains enriched with glycosphingolipids, most notably ganglioside GM1. GM1 exposes its glycan moiety to the lysosomal lumen, providing a scaffold for ligand binding. Aβ exhibits a high affinity for GM1, and Aβ42, in particular, undergoes conformational conversion and accelerated oligomerization on GM1 clusters [27]. Notably, PSEN1 localizes to raft-like membrane domains, and GM1 enhances PSEN1-dependent Aβ40/42 production, thereby increasing the plaque burden. Conversely, cholera toxin subunit B (CTB), which binds to GM1, blocks this interaction and mitigates Aβ-induced synaptic dysfunction in AD mouse models [28].

Pharmacological reduction in ganglioside synthesis using D-PDMP decreases GM1 levels, significantly reduces Aβ plaque deposition, and improves spatial learning and memory in APP/PS1 mice [29]. Mass spectrometry imaging studies have further revealed the co-localization of GM2 and GM3 with amyloid plaques in the cortex and hippocampus of aged APP/PS1 mice, suggesting that multiple gangliosides contribute to Aβ metabolism [30]. Therapeutic strategies targeting gangliosides in neurodegenerative diseases have been comprehensively reviewed elsewhere [31].

Beyond its role in γ-secretase activity, PSEN1 exerts γ-secretase-independent functions essential for lysosomal homeostasis. PSEN1 facilitates N-glycosylation and lysosomal targeting of the v-ATPase V0a1 subunit (a subunit predominantly expressed in the brain), thereby supporting lysosomal acidification [32,33,34]. Loss of PSEN1 function leads to impaired lysosomal acidification, although alternative studies argue that lysosomal dysfunction in PSEN-deficient cells arises primarily from disrupted Ca^2+^ homeostasis rather than proton pump failure [35]. The group that initially reported abnormal glycosylation of the V0a1 subunit subsequently demonstrated the disruption of Ca^2+^ homeostasis. This disturbance in calcium metabolism was shown to be secondary to impaired lysosomal acidification, as correction of lysosomal pH by the intralysosomal delivery of re-acidifying materials restored Ca^2+^ homeostasis [34]. In addition, congenital variants affecting the V0a1 subunit cause Developmental and epileptic encephalopathy-104 (DEE104 OMIM 619970), a disorder characterized by impaired intellectual development, microcephaly, and drug-resistant seizures. Brain biopsy findings in affected individuals have revealed swollen neurons and axons, with the accumulation of granular, PAS-positive deposits [36,37].

Taken together, these findings suggest that PSEN1 mutations, which broadly affect the post-translational modification of membrane proteins, including glycosylation and proteolytic processing, may promote AD through parallel mechanisms: impaired v-ATPase function due to reduced glycosylation of the V0a1 subunit, leading to lysosomal dysfunction and increased production of Aβ42 within the brain.

Notably, Aβ directly impairs lysosomal function. Recent studies using immunoprecipitation assays have demonstrated that intralysosomal Aβ binds to the v-ATPase V0c subunit (ATP6V0C), leading to lysosomal alkalization and subsequent autophagic dysfunction. Low-molecular-weight hyaluronic acid preserves dendritic spine density and cognitive performance by maintaining lysosomal function rather than altering Aβ uptake [38]. Thus, even in the absence of congenital PSEN1 mutations, progressive Aβ accumulation ultimately drives lysosomal neutralization in AD.

Paradoxically, Aβ oligomerization within lysosomes is promoted under acidic conditions in vitro, highlighting the dynamic and opposing interplay between Aβ aggregation and lysosomal pH regulation [26]. Failure of lysosomal acidification leads to organelle swelling and vacuolar accumulation, a pathology described as PANTHOS, which emerges before extracellular plaque formation [24]. Rupture of PANTHOS-laden neurons releases Aβ oligomers into the extracellular space, facilitating their propagation to neighboring cells.

Finally, intracellular Aβ42 not only impairs lysosomal function but also directly disrupts lysosomal membrane integrity, leading to cytosolic leakage in an in vivo assay [39]. The presence of apolipoprotein E4 further exacerbates Aβ42-induced lysosomal leakage and neuronal apoptosis [40].

Collectively, these findings support a model in which intracellular Aβ—particularly within the endo-lysosomal system—drives AD progression through reciprocal interactions with lysosomal structure and function. This perspective highlights novel therapeutic targets, including the disruption of GM1–Aβ interactions and preservation of v-ATPase-dependent lysosomal acidification as adjunct or alternative strategies beyond conventional extracellular Aβ targeting.

## 4. Tau Protein

### 4.1. Tau Pathobiology and the Lysosome–Cytoskeleton Axis in Alzheimer’s Disease

In 1986, multiple groups independently reported that tau is a major constituent of paired helical filaments (PHFs), the core ultrastructural component of neurofibrillary tangles (NFTs) [41,42,43]. As a microtubule-associated protein, tau contributes to microtubule stability and axonal transport, in part, through functional interactions with the kinesin- and dynein-dependent trafficking machinery [44]. Within the framework of the amyloid cascade hypothesis, AD can be conceptualized as an Aβ42-initiated tauopathy, implying a mechanistic coupling between Aβ and tau pathologies.

Consistent with such coupling, exposure to Aβ oligomers—particularly species formed under endo-lysosomal conditions—has been shown to induce tau missorting in cultured neurons, characterized by the redistribution of tau from axons to the somatodendritic compartment [26]. In primary neuronal cultures exposed to Aβ42, microtubule disruption, neurite beading, and aberrant localization of motor proteins, such as dynein and kinesin, have been observed, accompanied by organelle accumulation within the axons [45]. These transport failures contribute to the formation of dystrophic neurites surrounding plaques, thereby generating neuritic plaques and linking cytoskeletal collapse to canonical AD histopathology.

Given this cytoskeletal vulnerability, microtubule-stabilizing agents have been investigated as potential therapeutic agents [46]. Notably, the brain-penetrant small-molecule microtubule stabilizer CNDR-51997 was reported to safely reduce Aβ plaque pathology in 5XFAD mice and tau pathology in PS19 mice [47], supporting the feasibility of targeting microtubule integrity across amyloid- and tau-driven model systems.

### 4.2. Phospho-Rab Signatures, LRRK2 Activity, and Granulovacuolar Degeneration Bodies

Rab10 and Rab12 are members of the Rab family of small GTPases that regulate endosomal trafficking and receptor turnover [48,49]. In AD brain tissue, phosphorylated Rab10 has been reported to localize to senile plaques, NFTs, and granulovacuolar degeneration bodies (GVBs) [50], and phosphorylated Rab12 accumulates in NFTs and GVBs [51]. As both Rab10 and Rab12 are substrates of LRRK2 kinase, these observations suggest that LRRK2 activation is involved in the pathological milieu associated with NFT/GVB formation.

Importantly, phospho-Rab signals appear adjacent to, yet spatially separable from, AT8-positive tau within NFTs, and similar phospho-Rab accumulations have been observed across tauopathies, suggesting that these changes may not be uniquely Aβ-driven but instead reflect the upstream or downstream consequences of tau aggregation and associated organellar stress. Since LRRK2 activation is often interpreted as a response to lysosomal membrane perturbation, the presence of lysosome-associated phospho-Rabs in NFTs and GVBs supports a broader view in which lysosomal dysregulation is tightly coupled with tau pathology.

Pathologically, GVBs are commonly identified by immunoreactivity for casein kinase 1δ/ε and also for CHMP2B, an ESCRT-III component [52]. Although the ESCRT machinery is multifunctional, it plays a relevant role in lysosomal repair. Together with the concept that GVBs are lysosome-associated structures, these findings provide circumstantial support for a lysosome-centered mechanism that contributes to tau aggregation and neuronal degeneration.

In this context, a recent study using transdifferentiated neurons generated from fibroblasts of sporadic, late-onset AD patients reported elevated intracellular Aβ and tau levels compared with controls, along with proteostasis and lysosomal repair deficits [53]. The same study described a moderate correlation between intracellular Aβ burden and the number of CHMP2B puncta, as well as galectin-3 puncta, another marker of lysosomal damage, suggesting that Aβ accumulation and lysosomal injury are linked at the cellular level. Notably, the lysosome-targeting small molecule C381, reported to restore lysosomal acidification and resilience [54], normalized lysosomal vulnerability in these cells.

### 4.3. Vicious Cycles Linking Tau Accumulation to Autophagy–Lysosome Fusion Failure

Cytosolic tau accumulation is driven not only by tau detachment and missorting from microtubules [26], but also by self-reinforcing feedback loops that impair autophagic flux. Liu et al. proposed at least two tau-driven “vicious cycles” converging on the suppression of autophagosome–lysosome fusion.

First, tau accumulation represses the expression of IST1, a regulator linked to ESCRT-III complex formation, thereby inhibiting the autophagic flux in MAPT-expressing cultured cells. Mechanistically, tau was proposed to reduce histone acetylation and consequently downregulate IST1 [55]. Second, in a cellular experiment, tau accumulation induces ISG15 upregulation, which represses HDAC6 activity, leading to increased cortactin acetylation and impaired autophagosome–lysosome fusion [56]. In both scenarios, impaired tau clearance accelerates autophagosome accumulation and reinforces proteostatic stress. Tau-directed therapeutic strategies have been reviewed elsewhere [57] and are not discussed in detail here.

### 4.4. Asparaginyl Endopeptidase (AEP/Legumain/δ-Secretase) as a Convergent Driver of Aβ and Tau Pathology

Asparaginyl endopeptidase (AEP), originally identified in legumes and also known as legumain, is a lysosomal cysteine protease that has gained attention as a δ-secretase in AD. AEP cleaves tau at N255 and N368, generating toxic fragments, and recombinant APP at N585, thereby facilitating subsequent β- and γ-secretase processing and promoting Aβ production [56]. In APP-expressing HEK293 cells, AEP knockout reduces Aβ generation, and genetic deletion of AEP in AD mouse models (including 5XFAD and APP/PS1) decreases Aβ deposition [58].

AEP also indirectly modulates tau phosphorylation. By cleaving and activating the PP2A inhibitor I2PP2A, AEP suppresses protein phosphatase 2A activity, thereby enhancing tau hyperphosphorylation [59,60]. Through these convergent effects on both the APP/Aβ and tau pathways, AEP acts as an amplifier of AD pathology.

Under physiological conditions, AEP is primarily lysosomal and activated in acidic environments, which would, in principle, restrict its substrates to the endo-lysosomal compartment. However, when lysosomal membranes are compromised, such as in the setting of Aβ- or tau-induced lysosomal injury, AEP may leak into the cytosol, where it can cleave nearby tau and activate tau-phosphorylating cascades. This possibility is supported by recent evidence that intraneuronal Aβ accumulation can drive tau hyperphosphorylation via endolysosomal leakage [61].

Genetic ablation as well as pharmacological inhibition of AEP has been reported to improve cognitive function in mouse models of AD [58,62]. Administration of an AEP inhibitor (compound 11; δ-secretase inhibitor 11) to P301S and 5XFAD mice reduced the pathological processing of tau and APP and preserved their memory performance. Given that AEP expression in the brain is relatively low compared with that in several peripheral tissues, it has been argued that lower inhibitor doses may achieve therapeutic benefits while limiting systemic adverse effects, and potential applications have been proposed beyond AD, including traumatic brain injury and glioblastoma [63].

## 5. APOE4

Apolipoprotein E (APOE) is endocytosed via multiple receptors—including LDLR, LRP1, and ApoER2—and, after dissociating from the LDL receptor in the lysosomal lumen, facilitates lipid transfer from internalized lipoprotein particles to the lysosomal membrane through the action of TTYH2 (Tweety homologue 2) [64]. In the brain, astrocyte-derived APOE predominantly exists as three major isoforms (ε2, ε3, and ε4) [65]. Among these, APOE4 has been identified as a major genetic risk factor for late-onset AD, with a clear gene-dose effect [66].

Consistent with human genetics, humanized APOE knock-in mouse models have demonstrated isoform-dependent regulation of Aβ accumulation, with APOE4 knock-in animals exhibiting a greater Aβ plaque burden than their APOE3 counterparts [67]. Mechanistically, a synthetic peptide corresponding to the Aβ region implicated in APOE binding (amino acids 12–28) blocks the APOE–Aβ interaction; administration of this peptide to APOE ε2 or ε4 targeted replacement AD model mice reduced brain Aβ levels, insoluble APOE, and neuritic plaques, with a more pronounced effect in APOE4 mice [68].

In addition, experiments using cells expressing APOE4 have shown that APOE4 is associated with lysosomal alterations, suggestive of impaired function, including lysosomal enlargement and autophagosome accumulation. These findings raise the possibility that some of the lysosomal abnormalities observed in AD may, at least in part, be attributable to APOE4 [69]. Consistent with this interpretation, another group demonstrated that lysosomes in APOE4-expressing Neuro-2a cells exhibited significantly higher luminal pH than those in cells expressing APOE3, further supporting the association between APOE4 and lysosomal dysfunction [70].

Beyond AD, numerous APOE variants have been described as causes of familial type III hyperlipoproteinemia and lipoprotein glomerulopathy [71]. Notably, a hyperlipidemia-associated APOE4 variant, apoE4[L28P] (APOE4 Freiburg/Pittsburgh), was later shown to confer higher AD risk than wild-type APOE4 [72,73]. Conversely, rare C-terminal variants affecting the lipid-binding region, V236E and R251G, have been associated with approximately halved risk of AD relative to APOE ε3/ε3 [74]. Furthermore, in an individual carrying a PSEN1 mutation, homozygosity for APOE3 Christchurch (R136S) was associated with striking resistance to autosomal dominant AD, characterized by relatively limited tau accumulation on PET imaging despite substantial Aβ deposition [75]. The APOE3(V236E) variant exhibits a lower propensity for oligomer formation than APOE3 and has been shown to enhance cellular cholesterol efflux. In 5xFAD mice expressing APOE3(V236E), reductions in Aβ deposition, immune responses, and neuritic dystrophy were observed [76].

In addition, both APOE3(V236E) and APOE4(R251G) have been reported to display reduced structural stability compared to APOE3 and APOE4, respectively, and are more prone to unfolding. The variants R136S, V236E, and R251G have also been shown to increase the viability of cultured SK-N-SH cells and to reduce TNF-α secretion from BV2 microglia [77].

However, the biological properties and mechanisms of action of these protective variants remain incompletely understood, and further investigation is required to clarify their roles in AD pathogenesis and their potential therapeutic implications.

Collectively, these findings have motivated the therapeutic proposition that reducing APOE4 levels in carriers could be a rational disease-modifying strategy [72]. Key questions include the optimal timing of intervention, feasibility of sustained long-term treatment, and potential trade-offs, particularly given the evidence that APOE4 may be protective against age-related macular degeneration. In this context, a clinical trial employing an AAV vector to drive CNS expression of APOE2 (a putatively protective isoform) is ongoing (NCT03634007).

## 6. Microglia and Astrocytes

### 6.1. Complement Cascade as a Therapeutic Axis

In AD, a microglial subset often termed disease-associated microglia (DAM), engages in the phagocytosis of Aβ and lipids and is thought to constrain disease progression [78]. More specifically, DAM represent a distinct microglial population that was newly identified by single-cell RNA sequencing of microglia derived from 5XFAD mice [79] and has been observed in neurodegenerative diseases, including AD and amyotrophic lateral sclerosis. During the transition from the basal state of homeostatic microglia, the expression of genes characteristic of the homeostatic phenotype, such as *P2ry12*, *P2ry13*, *Cx3cr1*, and *Tmem119*, was downregulated. Simultaneously, the expression of genes such as *APOE* and *TYROBP/DAP12* was upregulated. Subsequently, in a second step that depends on TREM2 signaling, further activation occurs, accompanied by increased expression of genes such as *Cst7*, *Lpl*, and *Trem2*, resulting in the acquisition of the DAM phenotype. This two-step transition highlights the central role of TREM2-dependent signaling in microglial activation during neurodegeneration [79].

The observation that loss-of-function TREM2 variants, including R47H, constitute major AD risk factors after APOE4 [80,81] has been interpreted as genetic support for the protective role of TREM2-dependent microglial responses. TREM2 functions as a receptor for lipoprotein particles containing apolipoproteins, such as APOE and clusterin, thereby mediating the uptake of these lipoproteins by microglia. Aβ is associated with such lipoprotein particles and can be internalized together with them. In the presence of TREM2 variants, this uptake is impaired, leading to reduced microglial clearance of Aβ in vitro [82].

Notably, not only APOE isoforms and variants but also clusterin variants have been associated with the risk of AD [83]. Furthermore, when Aβ bound to APOE3-containing lipoproteins, which are relatively enriched in negatively charged phospholipids, was injected into the mouse brain, microglial activation occurred more rapidly than that with APOE4-containing lipoproteins. In addition, microglial barrier formation surrounding senile plaques in mouse models was more prominent in the presence of APOE3 lipoproteins. This difference has been attributed, at least in part, to the incomplete transition of microglia to the DAM state in the presence of APOE4 [84].

TREM2 physiologically functions as a multimer; however, molecular simulation studies have demonstrated that Alzheimer’s disease-associated TREM2 variants can interfere with multimer formation [85]. Furthermore, structural analyses of the TREM2–APOE3 complex have shown that the TREM2 R47H variant exhibits a reduced binding affinity for APOE3, whereas the TREM2 L69D variant shows a more pronounced reduction. In contrast, APOE4 displays a slightly higher affinity for wild-type TREM2 than APOE3 [86]. As supported by these molecular calculations, alterations in both TREM2 and APOE, two key regulators of Aβ uptake and handling, are closely linked to the pathogenesis of AD.

However, microglia can also contribute to pathology through synaptic pruning mechanisms. In rodent AD models, intrahippocampal administration of Aβ40 fibrils activates astrocytes and reduces glutamate transporter 1 (GLT1/EAAT2), thereby elevating synaptic glutamate levels [87]. In parallel, stimulation of the hippocampal CA1 metabotropic glutamate receptor 1 (mGluR1) increases synaptic C1q expression in AD rodent models [88]. Complement components, including C1q, localize to senile plaques, as demonstrated in early neuropathological studies conducted in the 1980s [89,90]. Functionally, intracerebroventricular delivery of soluble Aβ oligomers induces C1q deposition at synapses and reduces synaptic integrity in AD mouse models, as assessed by decreased co-localization of presynaptic synapsin and postsynaptic PSD95, within 72 h. Importantly, this synapse loss is C1q-dependent and can be prevented by anti-C1q antibodies [91].

Astrocyte–microglial coupling appears to modulate this complement-mediated pruning axis. Ceftriaxone, which upregulates GLT1, reduces C1q production and decreases microglial synapse engulfment, whereas pharmacological GLT1 inhibition increases C1q expression and enhances microglial pruning in AD rodent models [87]. These mechanisms align with neuropathological evidence that synaptic loss occurs early in AD and in mild cognitive impairment [92,93].

When Aβ is recognized by C1q, the classical complement pathway is activated, leading to C3 cleavage into C3b and further into iC3b formation. iC3b can engage microglial complement receptor 3 (CR3), promoting phagocytic responses reminiscent of developmental synaptic pruning and amplifying inflammatory signaling [91]. The therapeutic development of humanized anti-C1q antibodies (e.g., ANX005) is ongoing.

Downstream, C3b-driven C5 cleavage generates C5a and C5b. C5a activates C5aR1 expressed by plaque-associated microglia, thereby promoting neuroinflammation. Pharmacological antagonism of C5aR1 with the cyclic hexapeptide PMX205 has been reported to attenuate synapse loss in Tg2576 mice, and recent studies further support that C5aR1 signaling drives region- and age-dependent synaptic pruning and inflammatory glial programs in AD models [94,95]. For a broader perspective on immunomodulatory therapeutics for AD, refer to Duggan et al. [96].

### 6.2. Low-Intensity Pulsed Ultrasound as a Modulator of Glial States

Low-intensity pulsed ultrasound (LIPUS) has been reported to modulate intracellular signaling pathways, including the suppression of the PI3K/AKT pathway. This effect has been associated with reduced expression of inflammatory cytokines, including IL-1β and IL-6, in the mouse brain, accompanied by attenuation of microglia-mediated neuronal injury [97].

On the other hand, inhibition of PI3K/AKT signaling may lead to reduced activation of mTORC1 on lysosomes, which in turn promotes lysosomal acidification and enhances lysosomal activity [98]. From this perspective, as part of the mechanism, LIPUS may directly counteract lysosomal dysfunction, which has been proposed as a fundamental component of the pathophysiology of AD.

In a study using mutant tau-expressing mice (P301L), the application of LIPUS to one hemisphere resulted in increased blood–brain barrier permeability. However, no reduction in tau protein levels was observed in the stimulated hemisphere; instead, a non-significant trend toward increased tau accumulation was observed in some cases. Expression of the microglial activation marker Iba1 was reduced on the stimulated side in P301S mice, whereas in wild-type mice the opposite pattern was observed, with lower expression on the non-stimulated side [99].

In vivo, in mice receiving intraperitoneal LPS, impairments in the Morris water maze and novel object recognition tests were reported, with improvement trends following LIPUS stimulation. Concurrently, hippocampal and cortical expression changes in Aβ, APP, and caspase-3 have been reported to be normalized. In a human randomized, double-blind, placebo-controlled pilot trial, LIPUS-treated participants showed a trend toward reduced worsening of ADAS-J cog scores at 72 weeks, warranting further evaluation [100]. The rationale and design of this pivotal trial have been described recently [101].

## 7. Combination Therapies

Except in familial cases, the diagnosis of AD in most patients is initially based on clinical and imaging findings, while confirmation of underlying amyloid pathology often requires biomarker evidence such as amyloid PET imaging. Therapeutic approaches aimed at suppressing Aβ production alone may have limited efficacy once substantial amyloid deposition has occurred. In this context, antibody-based therapies capable of removing accumulated Aβ have attracted considerable attention.

Because the pathological stage of AD may differ across brain regions, the dominant molecular mechanisms—and therefore the most appropriate therapeutic targets—are also likely to vary spatially and temporally during disease progression. This consideration has led to an increasing interest in therapeutic strategies targeting multiple pathways rather than a single mechanism.

A Phase 2 clinical trial of the anti-tau antibody etalanetug (E2814) (NCT06602258) is being conducted in combination with lecanemab, reflecting ongoing efforts to evaluate combination therapies that address both amyloid and tau pathologies. In addition, inhibitors of asparagine endopeptidase (AEP), which may influence multiple pathogenic pathways, represent potential candidates for use in combination regimens. Combinations of such agents with disease-modifying antibodies, or alternatively with modalities such as LIPUS, may warrant further investigation, although their efficacy and safety remain to be established in future experimental and clinical studies.

## 8. Future Directions

The observation that TREM2 and PSEN1 variants predispose individuals specifically to AD rather than to other tauopathies, and that APOE isoforms and variants strongly influence the risk of AD, suggests that these genetic factors primarily affect the production, clearance, or accumulation of Aβ rather than tau itself. As described in the Thal Phases of Aβ deposition begins in the neocortex and subsequently spreads through subcortical regions, eventually reaching the brainstem and cerebellum [102]. Together with the relatively stereotyped pattern of tau propagation described by the Braak staging system, this reproducible spatial progression of pathology contributes to the clinical and pathological definition of AD as a distinct entity. However, the mechanisms underlying the highly stereotyped Aβ accumulation patterns remain unclear. Moreover, although tau pathology is influenced by Aβ in AD, it remains difficult to fully explain why tau accumulation appears to progress in a direction opposite to that of amyloid plaque deposition, spreading from the brainstem and limbic regions to the neocortex. Another unresolved issue is why, in contrast to many other tauopathies that predominantly involve 4-repeat tau, both 3-repeat and 4-repeat tau accumulate in AD, and how Aβ influences this isoform-specific pathology.

Although this mini-review is primarily concerned with AD, insights gained from comparisons with other tauopathies may be informative for understanding disease-specific mechanisms. Tuberous Sclerosis Complex Associated Neuropsychiatric Disorders (TAND) is a congenital condition caused by hyperactivation of mTOR and is characterized by neuronal accumulation of both 3-repeat and 4-repeat tau in the absence of Aβ deposition [103,104]. Because mTORC1 activation suppresses v-ATPase function [98], this mechanism partially overlaps with the pathways implicated in AD. However, TAND exhibits pathological features and lesion distributions that differ from those of AD. Notably, several clinical trials of rapamycin (sirolimus), an mTORC1 inhibitor used in the treatment of Tuberous Sclerosis Complex, are currently underway for AD (NCT04629495, NCT06022068). Niemann–Pick disease type C (NPC), caused by variants in NPC1 that lead to lysosomal cholesterol accumulation, shows neuropathological features more closely resembling AD, including the presence of amyloid plaques and neurofibrillary tangles [105]. PSEN1 has been shown to be involved not only in the glycosylation of the v-ATPase V0a1 subunit but also in the glycosylation of NPC1 [106]. A comparative consideration of TAND and NPC raises the possibility that lysosomal cholesterol accumulation may be a key determinant in the formation of amyloid plaques.

In vitro studies have suggested that tau protein may undergo liquid–liquid phase separation and form liquid droplets before fibrillar aggregation. However, it remains uncertain whether such liquid droplets form in degenerating neurons in vivo, whether pretangles represent a phase-separated state, and whether these processes are related to GVBs. Clarifying these questions may provide important insights into the early stages of tau aggregation. Despite these circumstances, recent in vitro studies have reported several molecular chaperones that co-condense within tau-containing liquid droplets and appear to suppress or delay tau fibril formation. These findings raise the possibility that if fibrillization can be slowed, the progression of tau pathology might be mitigated to some extent, even in the presence of impaired lysosomal function [107,108].

## 9. Conclusions

Collectively, the evidence reviewed here indicates that AD does not arise from a single linear pathogenic cascade but rather from the interplay of multiple molecular and cellular systems, including lipid metabolism, proteostasis, innate immunity, and endo-lysosomal homeostasis. APOE isoform-dependent lipid handling, Aβ and tau metabolism, ganglioside-rich membrane microdomains, v-ATPase-mediated lysosomal function, and glial responses converge on the endo-lysosomal network, which acts as a central hub linking extracellular pathology to intracellular degeneration. Disruption of this system, whether driven by Aβ accumulation, tau aggregation, APOE4-associated lipid dysregulation, or complement-mediated synaptic remodeling, appears to represent a shared vulnerability underlying the progression of the disease.

In discussing AD and its treatment, additional concepts such as γ-secretase modulators and liquid–liquid phase separation of tau protein are also highly relevant but are beyond the scope of this review. Nevertheless, AD represents a highly complex pathological state involving numerous players, including APP/Aβ, tau, APOE, gangliosides, v-ATPase, and microglia-associated proteins. From a disease-modifying perspective, anti-amyloid antibodies have already entered clinical practice, and clinical trials of anti-tau antibodies are underway. The multiplicity of molecular contributors implies the existence of multiple therapeutic intervention points, offering broad opportunities for drug development. As combination strategies targeting different aspects of the disease process are likely to be required, an increasingly detailed mechanistic understanding of AD pathophysiology will be essential for the rational design of future therapies.

## Data Availability

No new data were created or analyzed in this study.
